# Increased mortality in patients with non cystic fibrosis bronchiectasis with respiratory comorbidities

**DOI:** 10.1038/s41598-021-86407-8

**Published:** 2021-03-29

**Authors:** Hayoung Choi, Bumhee Yang, Yun Jin Kim, Sooim Sin, Yong Suk Jo, Youlim Kim, Hye Yun Park, Seung Won Ra, Yeon-Mok Oh, Sung Jun Chung, Yoomi Yeo, Dong Won Park, Tai Sun Park, Ji-Yong Moon, Sang-Heon Kim, Tae-Hyung Kim, Ho Joo Yoon, Jang Won Sohn, Hyun Lee

**Affiliations:** 1 Division of Pulmonary, Allergy, and Critical Care Medicine, Department of Internal Medicine, Hallym University Kangnam Sacred Heart Hospital, Hallym University College of Medicine, Seoul, South Korea; 2 Division of Pulmonary and Critical Care Medicine, Department of Internal Medicine, Chungbuk National University Hospital, Chungbuk National University College of Medicine, Cheongju, South Korea; 3 Biostatistical Consulting and Research Lab, Medical Research Collaborating Center, Hanyang University, Seoul, South Korea; 4 Department of Internal Medicine, School of Medicine, Kangwon National University, Chuncheon, South Korea; 5 Division of Pulmonary, Allergy, and Critical Care Medicine, Department of Internal Medicine, Hallym University Kangdong Sacred Heart Hospital, Hallym University College of Medicine, Seoul, South Korea; 6 Division of Pulmonary, Allergy, and Critical Care Medicine, Department of Internal Medicine, Hallym University Chuncheon Sacred Heart Hospital, Hallym University College of Medicine, Chuncheon, South Korea; 7 Division of Pulmonary and Critical Care Medicine, Department of Medicine, Samsung Medical Center, Sungkyunkwan University School of Medicine, Seoul, South Korea; 8 Division of Pulmonary Medicine, Department of Internal Medicine, Ulsan University Hospital, University of Ulsan College of Medicine, Ulsan, South Korea; 9 Department of Pulmonary and Critical Care Medicine, Asan Medical Center, University of Ulsan College of Medicine, Seoul, South Korea; 10 Division of Pulmonary Medicine and Allergy, Department of Internal Medicine, Hanyang University College of Medicine, Seoul, South Korea

**Keywords:** Prognosis, Risk factors, Epidemiology

## Abstract

There are limited data regarding whether mortality is higher in patients with non cystic fibrosis bronchiectasis (bronchiectasis) than in those without bronchiectasis. Using 2005–2015 data from the Korean National Health Insurance Service, we evaluated hazard ratio (HR) for all-cause mortality in the bronchiectasis cohort relative to the matched cohort. The effect of comorbidities over the study period on the relative mortality was also assessed. All-cause mortality was significantly higher in the bronchiectasis cohort than in the matched cohort (2505/100,000 vs 2142/100,000 person-years, respectively; *P* < 0.001). Mortality risk was 1.15-fold greater in the bronchiectasis cohort than in the matched cohort (95% confidence interval [CI] 1.09–1.22); mortality was greatest among elderly patients (HR = 1.17, 95% CI 1.10–1.25) and men (HR = 1.19, 95% CI 1.10–1.29). Comorbidities over the study period significantly increased the risk of death in the bronchiectasis cohort relative to the matched cohort: asthma (adjusted HR = 1.20, 95% CI 1.11–1.30), chronic obstructive pulmonary disease (adjusted HR = 1.24, 95% CI 1.15–1.34), pneumonia (adjusted HR = 1.50, 95% CI 1.39–1.63), lung cancer (adjusted HR = 1.85, 95% CI 1.61–2.12), and cardiovascular disease (adjusted HR = 1.34, 95% CI 1.23–1.45). In contrast, there were no significant differences in the risk of death in patients without bronchiectasis-related comorbidities and the matched cohort, except in the case of non-tuberculous mycobacterial infection. In conclusion, all-cause mortality was higher in patients with bronchiectasis cohort than those without bronchiectasis, especially in elderly patients and men. Comorbidities over the study period played a major role in increasing mortality in patients with bronchiectasis relative to those without bronchiectasis.

## Introduction

Non cystic fibrosis bronchiectasis (hereafter referred to as bronchiectasis) is a chronic respiratory disease being frequently encountered in daily practice^1^. Patients with bronchiectasis often suffer from persistent respiratory symptoms, decreased quality of life, and recurrent respiratory infection^1^. As the prevalence of bronchiectasis^2–7^ and associated health costs^2,8,9^ is increasing in many countries, this disease is becoming an emerging threat to global health^10,11^. However, unfortunately, these expectations are based on studies performed mostly in Western countries^10,11^. Thus, despite the general consideration that the prevalence of bronchiectasis is higher in Asian countries than in most Western countries, the epidemiology of bronchiectasis in most Asian countries is still lacking.

Reducing the mortality from bronchiectasis is one of the most important treatment outcomes of the disease. To achieve this outcome, the national mortality rate of bronchiectasis needs to be determined. It is also crucial to know the relative risk of mortality according to the presence or absence of bronchiectasis. This information would help decide whether a health policy is urgently needed to reduce mortality associated with bronchiectasis. However, limited information is available regarding the nationwide mortality due to bronchiectasis^10^. Furthermore, there is no known national representative data on mortality in patients with bronchiectasis in most Asian countries, including South Korea.

Comorbidities are known to increase the risk of mortality in patients with bronchiectasis^12^. These results have been well demonstrated by studies on a large number of bronchiectasis patients in Western countries^12^. However, since these comorbidities are also risk factors of mortality in the general population, it is unclear whether the comorbidities lead to higher mortality in bronchiectasis patients than in those without bronchiectasis. Given that improving survival is one of the major goals in treating chronic diseases, identifying the comorbidities associated with high mortality in patients with bronchiectasis relative to those without bronchiectasis is very important. However, limited data are available regarding this issue, especially in the Asian population.

Consequently, our principal aim was to compare the all-cause mortality in patients with bronchiectasis and those without bronchiectasis in Koreans. We also evaluated whether bronchiectasis-related comorbidities are associated with an increased mortality in patients with bronchiectasis relative to those without bronchiectasis.

## Methods

### Study population

**Figure 1:**
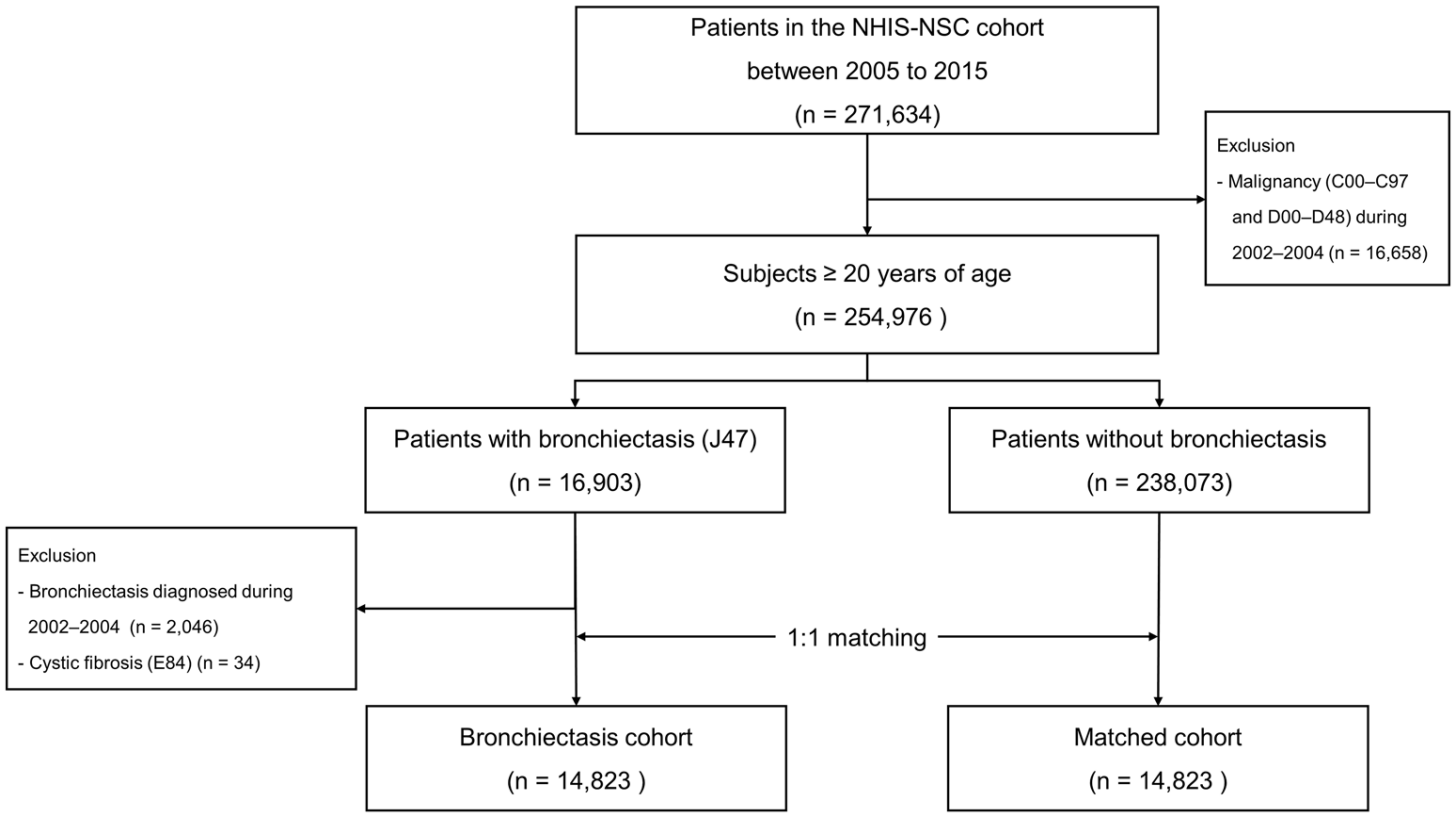
Flow chart of the study population. NHIS-NCS, National Health Insurance Service-National Sample Cohort.

This study used data from the National Health Insurance Service-National Sample Cohort (NHIS-NSC), which is a retrospective population-based cohort that includes 2.2% of all Korean citizens^13^. The NHIS-NSC collects data regarding major and minor diagnoses using the 10th revision of the International Statistical Classification of Diseases and Related Health Problems (ICD-10) codes, drug prescriptions, health examination outcomes, and mortality and the causes thereof^13^. As shown in Fig. 1, data regarding 271,634 patients aged ≥ 20 years were collected between January 2005 and December 2015. After exclusion of patients with any malignancy (ICD-10 diagnosis codes C00–C97 and D00–D48) during the washout period (January 2002–December 2004) (n = 16,658), 254,976 patients remained. Of these, 16,903 had bronchiectasis (ICD-10 diagnosis code J47). After the exclusion of patients with cystic fibrosis (ICD-10 diagnosis code E84) (n = 34) and patients diagnosed with bronchiectasis during washout period (January 2002–December 2004) (n = 2046), 14,823 incident bronchiectasis cases were identified. To establish a matched cohort, 1:1 matching was performed by age, sex, and Charlson Comorbidity Index (CCI) (baseline comorbidities were used to calculate CCI)^14^; accordingly, each patient with bronchiectasis was matched to a patient without bronchiectasis (Fig. 1)^15^. Patients were followed from the time diagnosed with bronchiectasis during the study period (January 2005–December 2015) until the date of death or until the end of the study period (December 31, 2015).

Methodologically, adult (aged ≥ 20 years) patients with bronchiectasis were included in this study because there are substantial differences between bronchiectasis in adults and that in children/adolescents^16^. Plus, we determined January 2002–December 2004 as the washout period and excluded patients with bronchiectasis during the period to include incident cases. The reason why we constructed an incident bronchiectasis cohort was that a prevalent cohort might underestimate short-term mortality because the more serious cases die in the early phase of the disease and are not included in the calculation of deaths over the period of observation^17^. For a similar reason, we excluded patients with malignancies during the washout period since the prevalent malignancies might overestimate the mortality of the study population.

The Institutional Review Board of Hanyang University Hospital approved the study and waived the requirement for informed consent because NHIS-NSC data are de-identified (approval no. HYUH 2019-05-020). All methods were performed in accordance with the relevant guidelines and regulations.

### Definition

Adult bronchiectasis was defined using the following criteria: (1) age ≥ 20 years and (2) at least one claim under ICD-10 code J47^6^. Bronchiectasis-associated comorbidities were defined using the following ICD-10 codes: angina pectoris (I20), myocardial infarction (I21, I22, or I25.2), asthma (J45–J46), chronic obstructive pulmonary disease (COPD) (J42–J44, except J43.0 [unilateral emphysema]), cerebrovascular disease (G45–G46, I60–I69, or H34.0), depression (F32–F34), diabetes mellitus (E10–E14), high cholesterol level (E78), gastroesophageal reflux disease (K21), hypertension (I10–I15), heart failure (I43, I50, I09.9, I11.0, I25.5, I13.0, I13.2, I42.0, I42.5–I42.9, or P29.0), inflammatory bowel disease (K50–K51), non-tuberculous mycobacteria (NTM) infection (A31), osteoporosis (M80–M81), and rheumatological disease (M05, M06, M31.5, M32, M33, M34, M35.1, M35.3, or M36.0)^15^. Baseline comorbidities were assessed at the time of study enrolment; those were used to calculate CCI (see Table 1 and Supplementary Table S1). Comorbidities over the study period were assessed at the time of study enrolment as well as during follow-up period. As we excluded patients with malignancies diagnosed during the washout period, lung cancer (C34) plus other malignancies (C00–C97, except C34) were only assessed during the follow-up period.

**Table 1:** Baseline patient characteristics.

		Bronchiectasis cohort (n = 14,823)	Matched cohort (n = 14,823)	*P* value
Age (years)	58.7 ± 15.0		58.7 ± 15.0	0.951
**Age group**				
20–29 years	590 (4.0)		587 (4.0)	1.0
30–39 years	1257 (8.5)		1255 (8.5)	
40–49 years	2073 (14.0)		2069 (14.0)	
50–59 years	3257 (22.0)		3268 (22.1)	
60–69 years	3709 (25.0)		3707 (25.0)	
≥ 70 years	3937 (26.5)		3937 (26.5)	
**Sex**				0.991
Male	7154 (48.3)		7153 (48.3)	
Female	7669 (51.7)		7670 (51.7)	
**Type of insurance**				
Self-employed health insurance	4216 (28.4)		5597 (37.8)	1.0
Employee health insurance	9347 (63.1)		9226 (62.2)	1.0
Medical aid	1260 (8.5)		–	
**Charlson Comorbidity Index**^a^	2.92 ± 2.5		2.92 ± 2.5	0.988

Data are presented as number (%) or mean with standard deviation.

^a^Comorbidities at the time of enrolment were used.

All-cause mortality was defined as all deaths during follow-up for up to 10 years after enrolment (January 2005–December 2015) irrespective of the cause of death. Causes of mortality were determined using data provided by Statistics Korea, an initiative of the Ministry of Strategy and Finance of South Korea. Causes of death were classified as one of the followings: (1) respiratory diseases (J00–J99); (2) cardiovascular diseases (I00–I99); (3) malignant neoplasms including lung cancer (C00–C97); (4) injury, poisoning, and external causes (S00–S99 and T00–T98); (5) endocrine diseases (E00–E90); (6) gastrointestinal diseases (K00–K93); (7) neurological diseases (G00–G99); (8) mental and behavioural disorders (F00–F99); (9) musculoskeletal and connective tissue diseases (M00–M99); and (10) miscellaneous.

### Outcomes

Primary outcomes were the comparison of overall mortality in the bronchiectasis cohort compared to the matched cohort. Secondary outcomes were the impact of bronchiectasis-related comorbidities on mortality in patients with and without bronchiectasis.

### Statistical analysis

The McNemar test was used to compare the baseline characteristics of the bronchiectasis and matched cohorts. The Kaplan–Meier method was used to generate survival curves, and the log-rank test was used to compare survival between the two groups. To evaluate the impact of bronchiectasis on mortality, mortality incidence rates (per 100,000 person-years) were compared between the bronchiectasis and matched cohorts using the normal approximation test for binomials. To determine hazard ratios (HR) for mortality, a Cox proportional hazards regression model was used, with adjustments for age, sex, insurance type, and CCI. For the evaluation of HR for each cause of mortality. In addition, to assess the effect of bronchiectasis-related comorbidities (asthma, COPD, pneumonia, NTM infection, lung cancer, and cardiovascular disease) on mortality in the bronchiectasis cohort versus the matched cohort, we used a Cox proportional hazard regression model with adjustment for age, sex, insurance type, and CCI. All statistical analyses were performed using SAS software ver. 9.4 (SAS Institute, Cary, NC, USA). All tests were two-sided and *P*-values < 0.05 were considered statistically significant.

## Results

### Baseline characteristics

The baseline characteristics of the bronchiectasis and the matched cohorts are summarised in Table 1. The mean age of the bronchiectasis cohort was 58.7 years, and 51.7% of the patients were women. There were no between-cohort differences in mean age, age group distribution, sex, insurance type, or CCI. Detailed comorbidity profiles at the time of study enrolment are summarised in Supplementary Table S1.

### Mortality of the bronchiectasis cohort relative to the matched cohort

**Figure 2:**
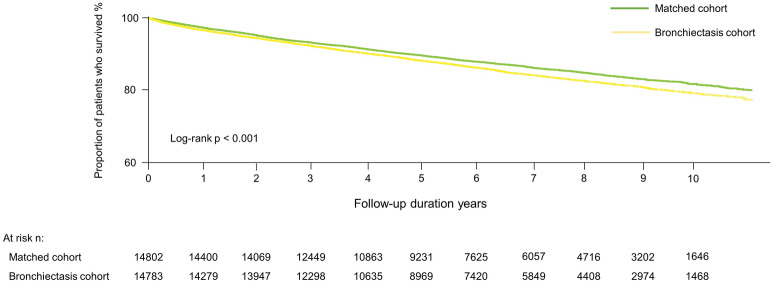
Kaplan–Meier survival analysis of time to death by bronchiectasis status.

As shown in Supplementary Figure S1, estimated all-cause mortality was significantly higher in the bronchiectasis cohort than in the matched cohort (2505.1/100,000 person-years vs 2142.2/100,000 person-years, *P* < 0.001), consistent with the results of survival analysis (Fig. 2). During the follow-up period, mortality risk was 1.15-fold greater (95% confidence interval [CI] 1.09–1.22) for patients in the bronchiectasis cohort than for those in the matched cohort. HRs for mortality in the bronchiectasis cohort relative to the matched cohort were 1.17 (95% CI 1.10–1.25) in elderly patients (age ≥ 60 years) and 1.19 (95% CI 1.10–1.29) in male. However, there were no significant differences in mortality risk in the bronchiectasis cohort relative to the matched cohort with respect to younger patients (age < 60 years) (HR = 1.17, 95% CI 0.98–1.39) or women (HR = 1.10, 95% CI 0.99–1.21) (Table 2).

**Table 2:** Mortality in the bronchiectasis cohort relative to the matched cohort.

	Total (N = 29,646)					Male (n = 14,307)					Female (n = 15,339)				
	No. at risk	No. of death	IR (/100,000 PY)	HR^a^	95% CI	No. at risk	No. of death	IR (/100,000 PY)	HR^a^	95% CI	No. at risk	No. of death	IR (/100,000 PY)	HR^a^	95% CI
**Overall**															
Matched	14,823	2058	2142.2	Ref	Ref	7153	1250	2781.3	Ref	Ref	7670	808	1580.4	Ref	Ref
BE	14,823	2250	2505.1	1.15	1.09–1.22	7154	1405	3362.0	1.19	1.10–1.29	7669	845	1759.5	1.10	0.99–1.21
**Age group**															
< 60 years															
Matched	7179	246	485.8	Ref	Ref	3398	168	713.6	Ref	Ref	3781	78	287.8	Ref	Ref
BE	7177	274	569.7	1.17	0.98–1.39	3394	195	872.2	1.22	0.99–1.51	3783	79	306.9	1.05	0.77–1.44
≥ 60 years															
Matched	7644	1812	3989.0	Ref	Ref	3755	1082	5056.4	Ref	Ref	3889	730	3038.3	Ref	Ref
BE	7646	1976	4736.5	1.17	1.10–1.25	3760	1210	6226.5	1.21	1.12–1.32	3886	766	3437.2	1.12	1.01–1.24

Data are presented as risk ratios (95% confidence interval).

^a^Unadjusted hazard ratio.

BE, bronchiectasis; IR, incidence rate; PY, person-years; HR, hazard ratio; CI, confidence interval; Ref, reference.

### Effects of bronchiectasis-related comorbidities on the risk of mortality in the bronchiectasis cohort relative to the matched cohort

**Figure 3:**
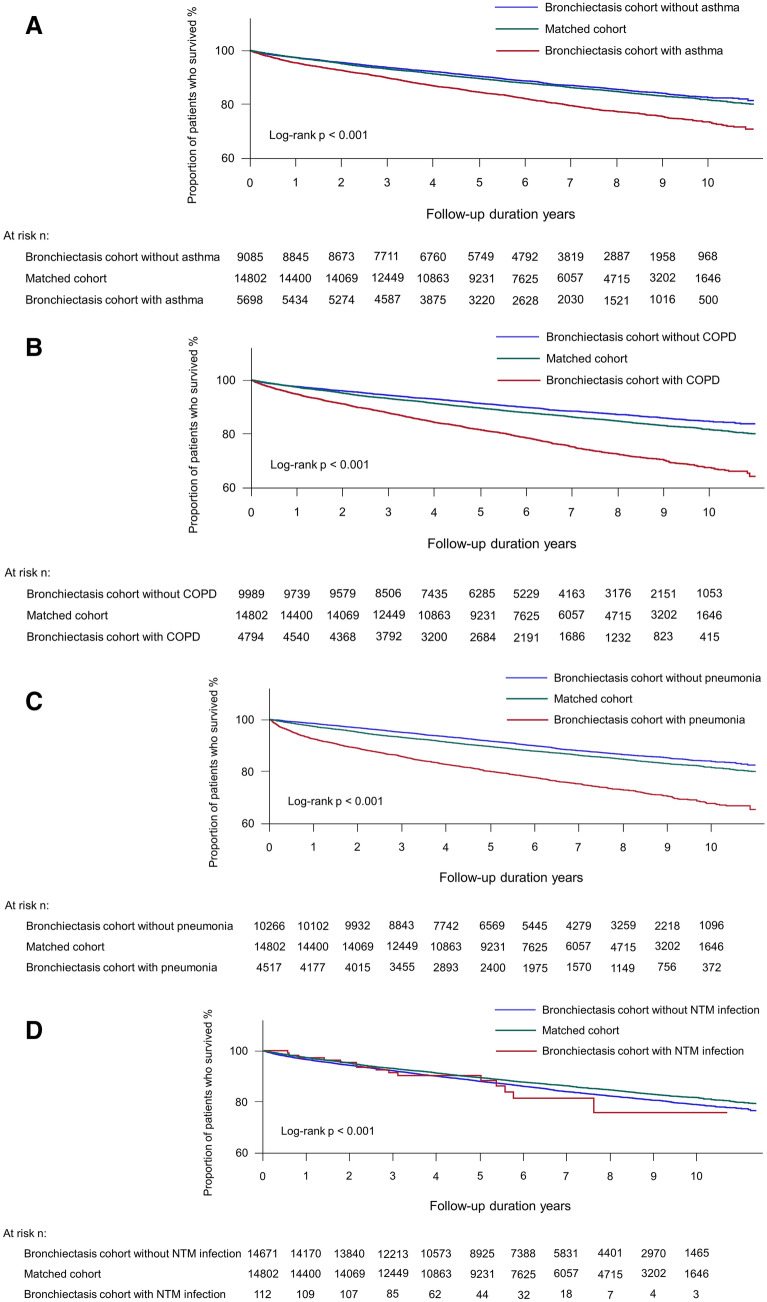

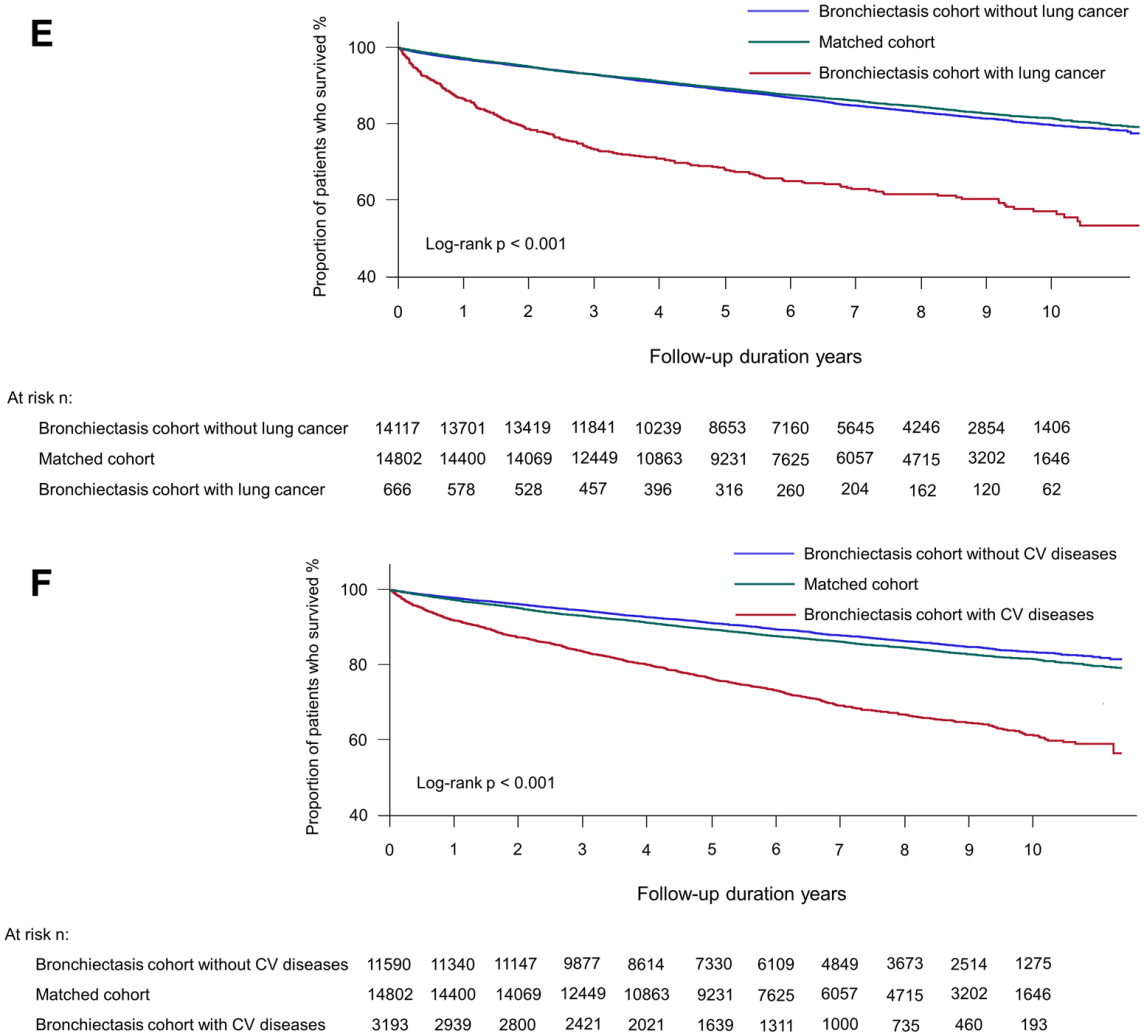
Kaplan–Meier survival analysis of the time to death in bronchiectasis patients with comorbidities, bronchiectasis patients without comorbidities, and those without bronchiectasis. (**A**) asthma, (**B**) COPD, (**C**) pneumonia, (**D**) NTM infection, (**E**) lung cancer, and (**F**) cardiovascular disease. COPD, chronic obstructive pulmonary disease; NTM, nontuberculous mycobacteria; CV, cardiovascular.

As shown in Table 3, the presence of the following bronchiectasis-related comorbidities—asthma (adjusted HR = 1.20, 95% CI 1.11–1.30), COPD (adjusted HR = 1.24, 95% CI 1.15–1.34), pneumonia (adjusted HR = 1.50, 95% CI 1.39–1.63), lung cancer (adjusted HR = 1.85, 95% CI 1.61–2.12), and cardiovascular disease (adjusted HR = 1.34, 95% CI 1.23–1.45)—significantly increased the risk of death in the bronchiectasis cohort relative to the matched cohort, in line with the survival analyses (asthma in Fig. 3A, COPD in Fig. 3B, pneumonia in Fig. 3C, lung cancer in Fig. 3E, and cardiovascular disease in Fig. 3F). However, patients with NTM infection in the bronchiectasis cohort did not have an increased risk of death compared to the matched cohort (adjusted HR = 1.07, 95% CI 0.64–1.78) (Fig. 3D).

**Table 3:** The effects of comorbidities over the study period on the risk of mortality in the bronchiectasis cohort relative to the matched cohort.

	Number at risk		Mortality		
		Number of death	Incidence rate (/100,000 PY)	Unadjusted HR (95% CI)	Adjusted HR^a^ (95% CI)
**Asthma**					
Matched cohort	14,823	2058	2142.2	Reference	Reference
Bronchiectasis cohort without asthma	9107	1149	2021.0	0.93 (0.87–1.00)	1.05 (0.97–1.13)
Bronchiectasis cohort with asthma	5716	1101	3337.4	1.53 (1.42–1.65)	1.20 (1.11–1.30)
**COPD**					
Matched cohort	14,823	2058	2142.2	Reference	Reference
Bronchiectasis cohort without COPD	10,015	1121	1795.6	0.82 (0.77–0.89)	1.02 (0.95–1.10)
Bronchiectasis cohort with COPD	4808	1129	4123.0	1.90 (1.77–2.04)	1.24 (1.15–1.34)
**Pneumonia**					
Matched cohort	14,823	2058	2142.2	Reference	Reference
Bronchiectasis cohort without pneumonia	10,283	1162	1793.5	0.83 (0.77–0.89)	0.91 (0.84–0.98)
Bronchiectasis cohort with pneumonia	4540	1088	4347.4	1.98 (1.84–2.13)	1.50 (1.39–1.63)
**NTM infection**					
Matched cohort	14,823	2058	2142.2	Reference	Reference
Bronchiectasis cohort without NTM infection	14,711	2235	2503.2	1.15 (1.08–1.22)	1.11 (1.05–1.19)
Bronchiectasis cohort with NTM infection	112	15	2843.6	1.29 (0.78–2.14)	1.07 (0.64–1.78)
**Lung cancer**					
Matched cohort	14,823	2058	2142.2	Reference	Reference
Bronchiectasis cohort without lung cancer	14,150	2009	2325.1	1.07 (1.01–1.14)	1.06 (0.99–1.13)
Bronchiectasis cohort with lung cancer	673	241	7069.1	3.18 (2.78–3.65)	1.85 (1.61–2.12)
**Cardiovascular disease**					
Matched cohort	14,823	2058	2142.2	Reference	Reference
Bronchiectasis cohort without cardiovascular disease	11,609	1342	1847.1	0.86 (0.80–0.92)	1.01 (0.94–1.08)
Bronchiectasis cohort with cardiovascular disease	3214	908	5290.9	2.40 (2.22–2.60)	1.34 (1.23–1.45)

Data are presented as risk ratios (95% confidence interval).

Comorbidities, including asthma, COPD, pneumonia, NTM infection, and cardiovascular disease, were assessed at the time of study enrolment as well as during the follow-up period; lung cancer was assessed during the follow-up period.

PY, person-years; HR, hazard ratio; CI, confidence interval; COPD, chronic obstructive pulmonary disease; NTM, non-mycobacterial mycobacteria.

^a^Adjusted for age, sex, type of insurance, and Charlson Comorbidity Index.

Patients in the bronchiectasis cohort without bronchiectasis-related comorbidities (asthma, COPD, pneumonia, lung cancer or cardiovascular disease) did not have an increased risk of death compared to the matched cohort, except in the case of NTM infection (adjusted HR = 1.11, 95% CI 1.05–1.19).

### Causes of mortality

The common causes of mortality among patients in the bronchiectasis cohort were malignant neoplasms, including lung cancer (29.7%); respiratory diseases (19.8%); cardiovascular diseases (17.8%); and injury, poisoning, and external causes (7.3%). In comparison, the common causes of mortality among patients in the matched cohort were malignant neoplasms (37.2%); cardiovascular diseases (21.9%); respiratory diseases (7.5%); and injury, poisoning, and external causes (7.0%).

## Discussion

We evaluated the impact of bronchiectasis on mortality using a longitudinal population-based cohort of Koreans. We found that patients with bronchiectasis were at increased risk of mortality compared to an age-, sex-, insurance type-, and CCI-matched population. The risks were highest in elderly patients and men. Bronchiectasis-related comorbidities (asthma, COPD, pneumonia, lung cancer, and cardiovascular diseases) could explain the increased risk of death in the bronchiectasis cohort relative to the matched cohort; however, patients in the bronchiectasis cohort without the comorbidities did not have increased risk of death compared to the matched cohort, except for NTM infection. The common causes of mortality in the bronchiectasis patients were malignant neoplasms, respiratory diseases, and cardiovascular diseases.

To the best of our knowledge, our study is the first to comprehensively analyse mortality in patients with bronchiectasis, compared to those without bronchiectasis, utilising a nationwide, representative, longitudinal, population-based cohort. Consistent with other authors, we found high mortality in patients with bronchiectasis^12,18–23^. However, in previous studies, the sample sizes were relatively small^18–22^, the mortality rate was not compared with that of controls^12,18–23^, or patients were only enrolled at a single centre^20–22,24^. Thus, data from previous studies were not generalisable. A cross-sectional study performed in the UK addressed these prior limitations by comparing mortality of bronchiectasis patients with that of the general population^10^. Although that analysis provided valuable insight regarding bronchiectasis mortality, the patients were not incident cases. Given the nature of a cross-sectional study, the study could only offer the mortality rate in a specified year in comparison with the general population^10^. Therefore, an important strength of our study was that we derived the bronchiectasis mortality rate by means of a large population-based longitudinal analysis of incident cases. Furthermore, we minimised selection bias by using nationally representative data.

We found that elderly patients and men with bronchiectasis were at higher risk of mortality than those without bronchiectasis. The analysis with the bronchiectasis cohort in this study revealed that older age^18,19,21,23,25^ and male sex^10,19,23^ were significantly associated with higher mortality, consistent with the findings in previous studies. Although the reasons for the elderly and male associations with poor outcomes are unclear, we may acquire insight from COPD mortality studies showing similar results. One study in the Netherlands found that mortality was higher for patients with COPD than for patients without COPD, especially among men and elderly patients^26^. A recent Korean study found an increasing HR trend for mortality in men with COPD compared to men without COPD^27^. Whether such findings are common among patients with chronic respiratory diseases is unclear. Further studies are needed.

Another interesting finding was that bronchiectasis-related comorbidities could explain the increased risk of death in the bronchiectasis cohort compared to the matched cohort, as the risk of death in bronchiectasis patients without bronchiectasis-related comorbidities was comparable to the risk of death in those without bronchiectasis. This finding suggests that bronchiectasis-related comorbidities, including asthma, COPD, pneumonia, lung cancer, and cardiovascular diseases, may play a major role in increased mortality in the bronchiectasis cohort relative to the matched cohort. As shown in the previous reports^18,21–24^, respiratory conditions were common causes of mortality among patients with bronchiectasis in the present study. Comorbid COPD and asthma (both of which are included in the Bronchiectasis Aetiology Comorbidity Index [BACI]) are significantly associated with higher mortality in patients with bronchiectasis^21,23,28–31^. In contrast, the bronchiectasis cohort without NTM infection showed increased mortality relative to the matched cohort; however, the bronchiectasis cohort with NTM infection did not show increased mortality relative to the matched cohort. This phenomenon may be explained by the fact that macrolide, a core drug used in NTM infection, exert a protective effect on the mortality of the bronchiectasis patients with NTM infection^32^. However, the number of bronchiectasis cohort with NTM infection was relatively small not enough to draw definite conclusions; future research is needed. Despite increasing evidence of higher mortality in patients with bronchiectasis who have other comorbid pulmonary diseases (compared to patients with bronchiectasis who do not have other comorbid pulmonary diseases), no evidence-based treatment strategy is yet available for such patients. Both our results and previous findings emphasise the urgent need for integrated treatment strategies for patients who have bronchiectasis and other pulmonary diseases.

Notably, we found that cardiovascular diseases (not included in the BACI) in bronchiectasis were associated with higher mortality in patients with bronchiectasis relative to those without bronchiectasis. Previous studies showed that approximately 20% of deaths among patients with bronchiectasis were of cardiovascular origin^21,23^. Because the risk of coronary heart disease and stroke is higher in patients with bronchiectasis than in the general population^33,34^, such comorbidities require close attention in patients with bronchiectasis. Although we excluded patients diagnosed with malignancies at the time of enrolment, malignancy (especially lung cancer) was an important cause of mortality in patients with bronchiectasis, as shown in a previous study^24^. When we consider that many bronchiectasis patients had COPD^35^, a well-known risk factor for lung cancer^36^, we suspect that bronchiectasis may increase the risk of lung cancer^37^. Hence, surveillance for lung cancer may be beneficial in patients with bronchiectasis who have risk factors for lung cancer, including a history of smoking, tuberculosis, or COPD.

Our study had several limitations. First, the study only included the evaluation of Korean patients; therefore, our data may not be generalisable to other ethnic groups or populations. Second, the study may not have included patients with bronchiectasis who had mild symptoms because the ICD-10 code was used for the diagnosis of bronchiectasis. Plus, given the nature of study using ICD-10 codes, some of the study population might have been misclassified as having bronchiectasis. Third, this study did not include mortality factors evaluated in prior studies (e.g., smoking status^21^, body mass index^18^, or microbiological data^21,38,39^) because the NHIS-NCS database lacks such data.

In conclusion, all-cause mortality was significantly higher in patients with bronchiectasis than in those without bronchiectasis, especially in elderly patients and men. Bronchiectasis-related comorbidities played a major role in increased mortality in the bronchiectasis cohort relative to the matched cohort.

## Supplementary Information

Supplementary Information.

## Data Availability

All data extracted in this study are included in the current article.
