# Supplementary material for: Increased mortality in patients with non cystic fibrosis bronchiectasis with respiratory comorbidities
Source: Sci Rep. 2021 Mar 29;11:7126. doi: 10.1038/s41598-021-86407-8 (PMC8007811; doi:10.1038/s41598-021-86407-8)

Increased mortality in patients with non cystic fibrosis bronchiectasis with respiratory comorbidities

Hayoung Choi[1], Bumhee Yang[2], Yun Jin Kim[3], Sooim Sin[4], Yong Suk Jo[5], Youlim Kim[6], Hye Yun Park[7], Seung Won Ra[8], Yeon-Mok Oh[9], Sung Jun Chung[10], Yoomi Yeo[10], Dong Won Park[10], Tai Sun Park[10], Ji-Yong Moon[10], Sang-Heon Kim[10], Tae-Hyung Kim[10], Ho Joo Yoon[10], Jang Won Sohn[10], Hyun Lee[10]

[1]Division of Pulmonary, Allergy, and Critical Care Medicine, Department of Internal Medicine, Hallym University Kangnam Sacred Heart Hospital, Hallym University College of Medicine, Seoul, Korea. [2]Division of Pulmonary and Critical Care Medicine, Department of Internal Medicine, Chungbuk National University Hospital, Chungbuk National University College of Medicine, Cheongju, Korea. [3]Biostatistical Consulting and Research Lab, Medical Research Collaborating Center, Hanyang University, Seoul, Korea. [4]Department of Internal Medicine, School of Medicine, Kangwon National University, Chuncheon, Korea. [5]Division of Pulmonary, Allergy, and Critical Care Medicine, Department of Internal Medicine, Hallym University Kangdong Sacred Heart Hospital, Hallym University College of Medicine, Seoul, Korea. [6]Division of Pulmonary, Allergy, and Critical Care Medicine, Department of Internal Medicine, Hallym University Chuncheon Sacred Heart Hospital, Hallym University College of Medicine, Chuncheon, Korea. [7]Division of Pulmonary and Critical Care Medicine, Department of Medicine, Samsung Medical Center, Sungkyunkwan University School of Medicine, Seoul, Korea. [8]Division of Pulmonary Medicine, Department of Internal Medicine, Ulsan University Hospital, University of Ulsan College of Medicine, Ulsan, Korea. [9]Department of Pulmonary

and Critical Care Medicine, Asan Medical Center, University of Ulsan College of Medicine, Seoul, Korea. [10]Division of Pulmonary Medicine and Allergy, Department of Internal Medicine, Hanyang University College of Medicine, Seoul, Korea.

Supplementary Table S1. Comparison of comorbidity profiles between the bronchiectasis cohort and the matched cohort at the time of study enrolment.

	**Bronchiectasis cohort (n=14,823)**	**Matched cohort (n=14,823)**	***P*-value**
**Pulmonary comorbidities**	4,366 (29.5)	4,575 (30.9)	0.009
COPD	1,733 (11.7)	1,337 (9.0)	<0.001
Asthma	2,427 (16.4)	2,228 (15.0)	0.002
Non-tuberculous mycobacterial infection	5 (0.03)	11 (0.1)	0.134
Pneumonia	951 (6.4)	853 (5.8)	0.017
**Extra-pulmonary comorbidities**			
Diabetes mellitus	2,227 (15.0)	4,096 (27.6)	<0.001
Hypertension	3,955 (26.7)	6,522 (44.0)	<0.001
Cardiovascular disease	1,523 (10.3)	2,478 (16.7)	<0.001
Angina	858 (5.8)	1,401 (9.5)	<0.001
Myocardial infarction	135 (0.9)	237 (1.6)	<0.001
Congestive heart failure	271 (1.8)	630 (4.3)	<0.001
Crohn's disease	25 (0.2)	13 (0.1)	0.051
Ulcerative colitis	32 (0.2)	33 (0.2)	0.901
Cerebrovascular diseases	927 (6.3)	1,875 (12.7)	<0.001
Nerve disorder	2,855 (19.3)	4,208 (28.4)	<0.001
Rheumatoid arthritis	671 (4.5)	792 (5.3)	0.001
Inflammatory bowel disease	55 (0.4)	46 (0.3)	0.370
Osteoporosis	1,146 (7.7)	1,823 (12.3)	<0.001
Reflux esophagitis	1,832 (12.4)	3,549 (24.0)	<0.001

Data are presented as number (%).
COPD, chronic obstructive pulmonary disease.

Supplementary Figure S1. All-cause mortalities.

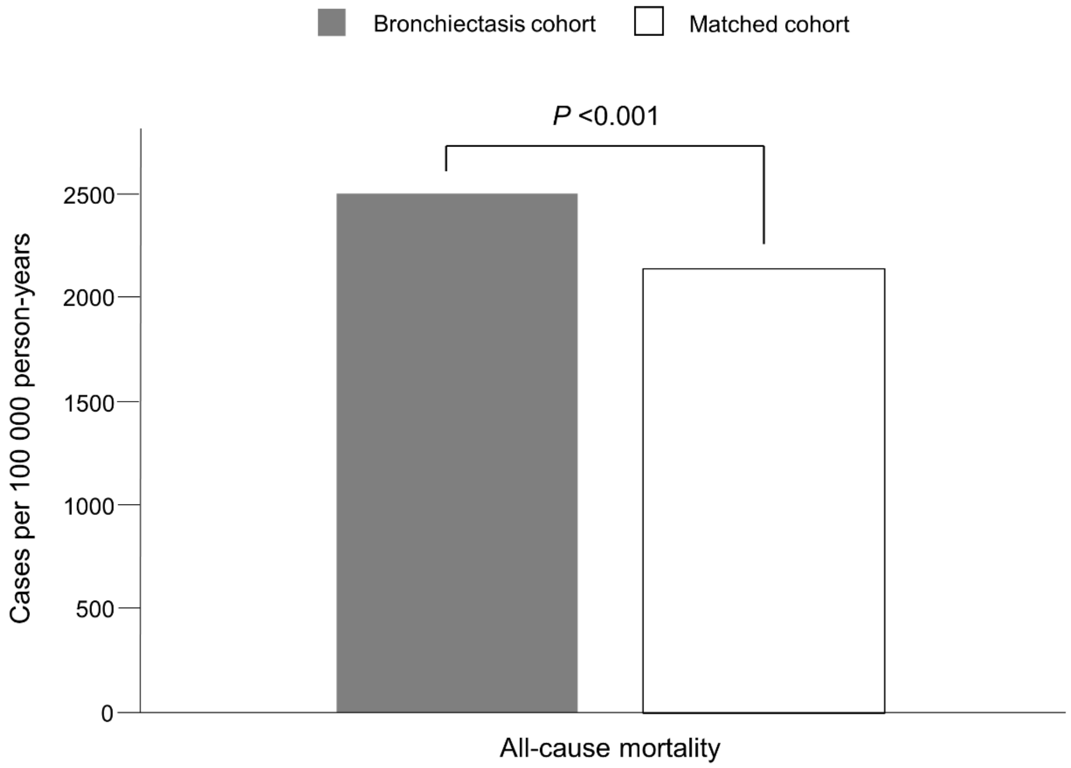

Supplement: Supplementary file 1 — Supplementary Information. [file 41598_2021_86407_MOESM1_ESM.pdf]
